# Deficiency of miRNA-149-3p shaped gut microbiota and enhanced dextran sulfate sodium-induced colitis

**DOI:** 10.1016/j.omtn.2023.01.011

**Published:** 2023-02-07

**Authors:** Esko Kankuri

**Affiliations:** 1 Department of Pharmacology, Faculty of Medicine, University of Helsinki, Helsinki, Finland

**Keywords:** mir-149, colitis, drug therapy, gut microbiota, IBD

Worldwide, inflammatory bowel disease (IBD) is estimated to affect 6.8 million individuals, and its prevalence is increasing.^1^ In countries with a lower socio-demographic index (SDI), the increase in prevalence is dominated by increases in IBD incidence. The high SDI countries, on the other hand, are undergoing a period of increased compounding prevalence, which is characterized by increased disease burden due to increased years lived with disease despite a steady IBD incidence.^1^^,^^2^ It has been estimated that by the year 2030, the number of people living with IBD will surpass 10 million.^2^

Widening of the therapeutic arsenal for IBD, as seen in many chronic inflammatory conditions, from glucocorticoids to biological therapies and small-molecule drugs, for example tumor necrosis factor α (TNF-α) and Janus kinase (JAK) inhibitors, has offered several treatment options to help personalize disease management. Although the current therapeutic regimens are far from optimal, due to their associated adverse effects, opportunities for fine-tuning and improving the individualization of IBD therapy are many. The search for robust biomarkers reflecting the level of mucosal healing without endoscopy will eventually help us to gain an understanding of the best combined use of drugs, their dose optimizations, and their therapy breaks. Moreover, gut-selective treatments or formulations resulting in low plasma exposure could rationally provide good mucosal anti-inflammatory efficacy with less adverse effects.

As our understanding of IBD pathogenesis continues to increase, the disease contribution of crosstalk between microbiota and the gut mucosa gains importance. For example, untargeted secretion of immunoglobulin A (IgA) through innate mechanisms contributes to the maintenance of microbiota diversity, while colitogenic bacteria are selectively targeted by adaptive, T cell-dependent mechanisms.^3^ Disruptions in this intricate interplay between the microbiota and the mucosal innate and adaptive responses lead to dysbiosis and can drive or aggravate disease. This interaction can be utilized for therapeutic benefit by targeting the mucosal and inflammatory cell activity or by modulating the microbiota. Results from a recent randomized clinical trial provided added proof to the latter and showed that fecal microbiota transplantation with dietary control may serve as an alternative to medical therapy in mild to moderate disease.^4^

Broad control over mucosal inflammation and pro-inflammatory signaling is achievable through inhibition of pro-inflammatory or activation of anti-inflammatory factors or pathways. MicroRNAs (miRNAs) widely modify cellular responses by binding to several RNA targets posttranscriptionally. They are short ∼22 nucleotide single strands of RNA, and although their actions are intricately regulated on various levels, canonically, they recognize complementary sequences in 3′ untranslated regions of mRNAs.^5^ At their most powerful, anti-inflammatory miRNAs can resemble glucocorticoids in their wide spectrum of actions to activate anti-inflammatory pathways and suppress pro-inflammatory ones. Although much of the microRNAs’ mechanism to regulate fate of specific mRNAs as well as the cell- and signaling-context dependency of their actions remain uncovered, they provide a crucial level of control to a variety of physiological processes. Moreover, their reduced or increased levels can contribute to the development and persistence of disease.

**Figure 1:**
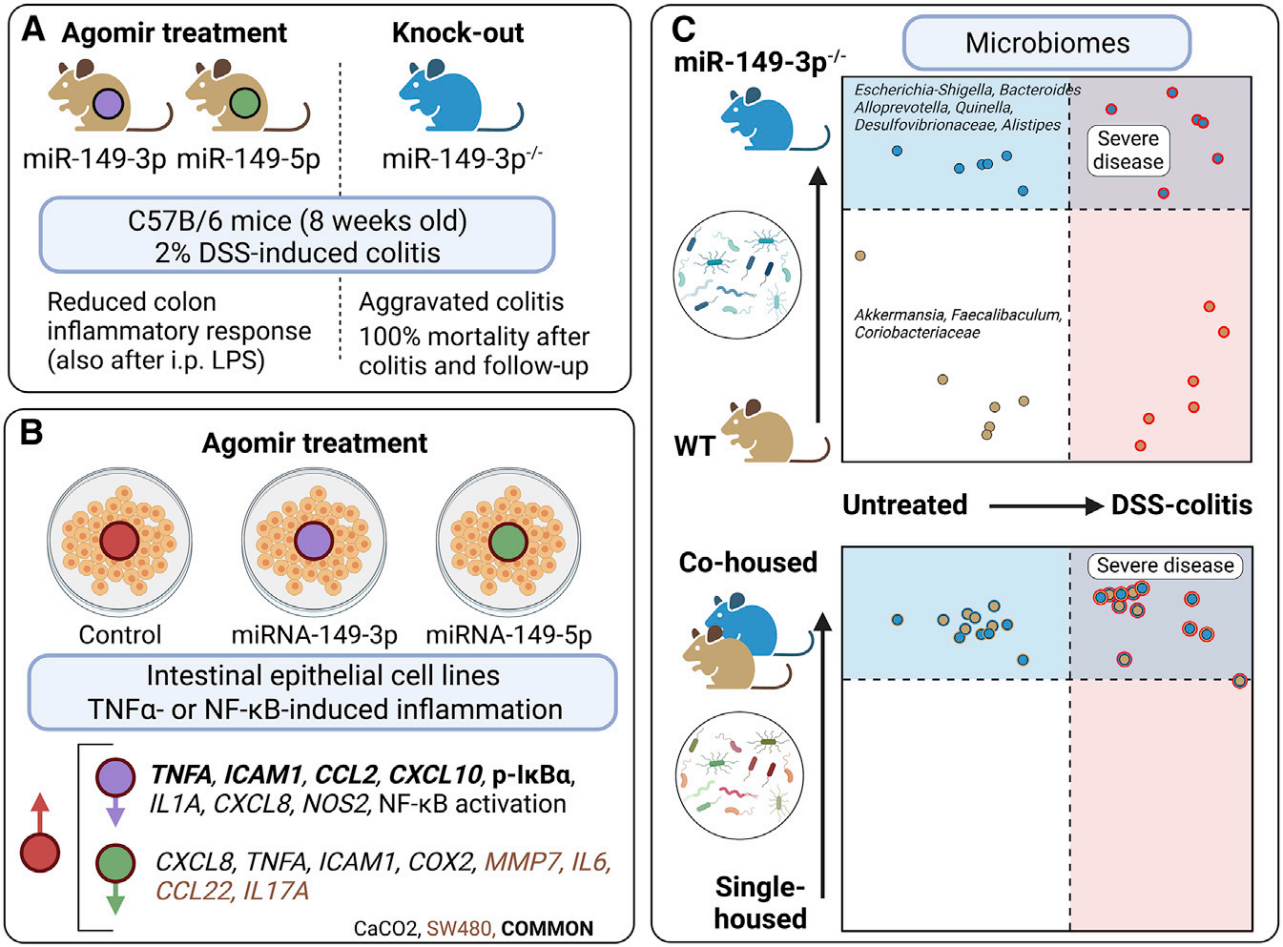
Representative composition of results of the Feng et al. study (A) DSS colitis in C56/B6 wild-type (WT) and *miR-149-3p*^−/−^ knockout (KO). (B) Effects of miR-149-3p and -5p agomirs on TNF-α-induced or NF-κB p65 overexpression-induced inflammation. (C) Effects of co-housing on microbiome with or without DSS colitis in WT and *miR-149-3p*^−/−^ KO mice.

In their report, Feng et al.^6^ assessed the role of miR-149-3p in an experimental model of murine dextran sulfate sodium (DSS)-induced colitis. A representation of their main findings is shown in Figure 1. The authors first demonstrated increased production of pro-inflammatory mediators in the colons of *miR-149-3p*^−/−^ knockout (KO) mice and found an aggravated colonic inflammatory response in these mice to DSS. Of note, after a period of 7 days with 2% DSS in their drinking water, followed up with a 7-day period on water, all *miR-149-3p*^−/−^ mice died, indicating severe uncontrollable and prolonged inflammatory reactivity. DSS-induced colitis of wild-type (WT) mice was alleviated by treatment with miR-149-3p or miR-149-5p commercial agomirs (MicrON, Ribobio, Guangzhou, P.R. China). *In vitro*, these agomirs suppressed TNF-α-induced cytokine secretion and p65 nuclear factor κB (NF-κB) activity. The authors then investigated microbiome changes induced by both *miR-149-3p*^−/−^ KO and colitis. Both miR-149-3p deficiency and colitis specifically modified the gut microbiome, and intriguingly, co-housing *miR-149-3p*^−/−^ and WT mice resulted in aggravated DSS-induced colitis in the WT mice. The susceptibility of the *miR-149-3p*^−/−^ KO mice to colon inflammation remained unchanged. Specifically, the microbiome of the WT mice was shifted to reflect that of the KO mice. Thus, the lack of miR-149-3p induced disease- and inflammation-promoting changes to the colon microbiota. However, even pretreatment of the KO mice with 4× antibiotics (vancomycin, ampicillin, kanamycin, and metronidazole) did not offer protection against DSS-induced colitis, suggesting susceptibility to colitis in these miR-149-3p-deficient mice not to be primarily caused by the microbiota.

Increased levels of microRNAs such as miR-16, miR-21, and miR-223 have been measured in intestinal biopsies and feces of individuals with IBD.^7^ As miRNAs regulate complex molecular networks and affect gene regulation on various levels, it is difficult to pinpoint their exact mechanism of action in a given pathology. For example, although miR-223 levels are increased in IBD, miR-223 agomir supplementation was associated with therapeutic activity. The miR-149 with anti-inflammatory activity, however, seems to demonstrate a more straightforward association in IBD. Its expression is decreased in blood samples of patients with Crohn’s disease, and it has been shown to downregulate Toll-like receptor-triggered inflammation and cytokine production through the MyD88 signal transduction adaptor and NF-κB signaling.^8^ The anti-inflammatory activity of miR-149 agomirs in inflammatory conditions such as psoriasis have also been demonstrated to occupy signaling through TWEAK receptor and interleukin-6 (IL-6).^9^

Recently, high expression of DLGAP1-AS1 in colorectal cancer (CRC) was shown to be correlated with metastasis and poor survival outcome. In CRC cell lines, such as SW480 and HT29 also used by Feng et al.,^6^ its expression was also increased.^10^ Interestingly, DLGAP1-AS1 was shown to downregulate mir-149-5p, the tumor-suppressive functions of which were characterized to include increased apoptosis and suppression of TGFB2 expression. Taken together with the results from Feng et al.,^6^ it would be interesting to evaluate if this DLGAP1-AS1 pathway is also involved in colitis and if it acts as a putative link between inflammation and cancer. Moreover, based on these results^6^ in general, it will be of great interest to understand if IBD therapeutics convey their therapeutic effects in IBD through modulation of miR-149. The results so far would indicate, e.g., that TNFα-, IL-6-, and JAK-inhibitors possibly affect this signaling pathway.

Lastly, it is important to note that the experimental colitis models, including DSS colitis, are unable to recapitulate all pathological aspects of human IBD. Therefore, it will be important to evaluate the effects of miR-149 agomirs in other IBD models to gain further understanding on their actual clinical therapeutic potential. Optimizing for gut selectivity and minimizing systemic exposure after oral administration could serve as the future aims for targeted IBD oligonucleotide therapeutics.
